# Possible Health Effects of a Wax Ester Rich Marine Oil

**DOI:** 10.3389/fphar.2020.00961

**Published:** 2020-06-26

**Authors:** Pauke Carlijn Schots, Alice Marie Pedersen, Karl-Erik Eilertsen, Ragnar Ludvig Olsen, Terje Steinar Larsen

**Affiliations:** ^1^ Faculty of Biosciences, Fisheries and Economics, Norwegian College of Fishery Science, UiT The Arctic University of Norway, Tromsø, Norway; ^2^ Calanus AS, Tromsø, Norway; ^3^ Cardiovascular Research Group, Department of Medical Biology, UiT The Arctic University of Norway, Tromsø, Norway

**Keywords:** *Calanus finmarchicus*, obesity, long-chain omega-3 fatty acids, long-chain monounsaturated fatty acids, long-chain fatty alcohol, stearidonic acid, cardiovascular diseases, inflammation

## Abstract

The consumption of seafood and the use of fish oil for the production of nutraceuticals and fish feed have increased over the past decades due the high content of long-chain polyunsaturated omega-3 fatty acids. This increase has put pressure on the sustainability of fisheries. One way to overcome the limited supply of fish oil is to harvest lower in the marine food web. *Calanus finmarchicus,* feeding on phytoplankton, is a small copepod constituting a considerable biomass in the North Atlantic and is a novel source of omega-3 fatty acids. The oil is, however, different from other commercial marine oils in terms of chemistry and, possibly, bioactivity since it contains wax esters. Wax esters are fatty acids that are esterified with alcohols. In addition to the long-chain polyunsaturated omega-3 fatty acids, eicosapentaenoic acid (EPA) and docosahexaenoic acid (DHA), the oil is also rich in stearidonic acid (SDA), long-chain monounsaturated fatty acids, and the long-chain fatty alcohols eicosenol and docosenol. Recent animal studies have indicated anti-inflammatory and anti-obesogenic actions of this copepod oil beyond that provided by EPA and DHA. This review will discuss potential mechanisms behind these beneficial effects of the oil, focusing on the impact of the various components of the oil. The health effects of EPA and DHA are well recognized, whereas long-chain monounsaturated fatty acids and long-chain fatty alcohols have to a large degree been overlooked in relation to human health. Recently, however the fatty alcohols have received interest as potential targets for improved health *via* conversion to their corresponding fatty acids. Together, the different lipid components of the oil from *C. finmarchicus* may have potential as nutraceuticals for reducing obesity and obesity-related metabolic disorders.

## Introduction

It is widely accepted that the omega-3 (n-3) long-chain polyunsaturated fatty acids (LC-PUFA) eicosapentaenoic acid (EPA, 20:5n-3) and docosahexaenoic acid (DHA, 22:6n-3), present in seafood, have health benefits in several human diseases and conditions, such as cardiovascular and inflammatory diseases. They also play a critical role in neural development (Campoy et al., 2012; Delgado-Lista et al., 2012; Calder, 2015). Responsible organizations, such as the World Health Organization, European Food Safety Authority and the American Heart Association, recommend, therefore, at least one or two servings of (oily) fish per week, equivalent to about 250 mg/day of EPA and DHA (WHO, 2003; EFSA, 2009; Rimm et al., 2018).

The use of fish oil in aquaculture feed and as a nutraceutical for direct human consumption has increased over the past decades (Tocher, 2015). This, in addition to the increased consumption of seafood, has put pressure on sustainable fisheries, and it has been estimated that about 33% of the world’s marine fish stocks are overfished (FAO, 2018). Still there is a gap between supply and demand for marine oils (Tocher, 2015; FAO, 2018) and therefore a need for new and sustainable sources of marine lipids. One possibility is to harvest lower in the marine food web. Zooplankton and small crustaceans like krill that feed on phytoplankton, the producers of the n-3 LC-PUFA, are to some degree now being utilized for production of marine oil nutraceuticals.

**Figure 1:**
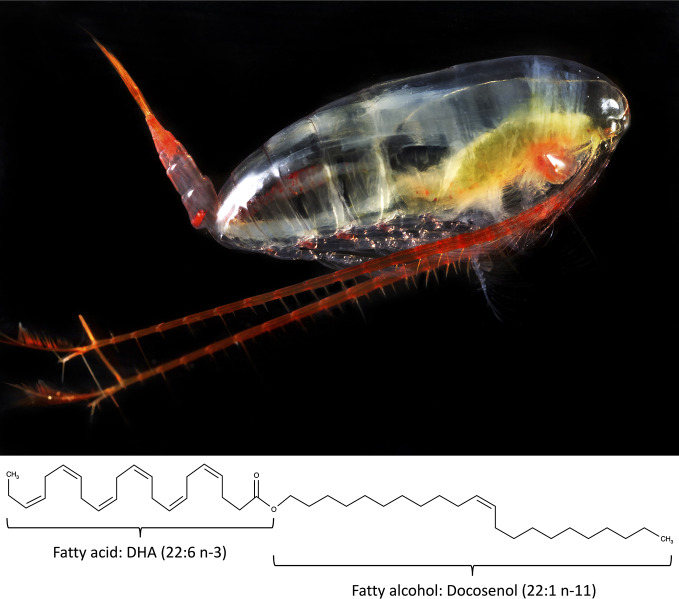
The marine copepod *Calanus finmarchicus* and a wax ester composed of the long-chain polyunsaturated omega-3 fatty acid docosahexaenoic acid (22:6 n-3) bound to the long chain fatty alcohol docosenol (22:1 n-11) as an example of the lipids found in the oil of *Calanus finmarchicus*. Photo Copyright by Calanus AS.

*Calanus finmarchicus* (*Cf*), a small marine copepod (**Figure 1**), constitutes a considerable proportion of the biomass in the Norwegian Sea (Planque and Batten, 2000) and is currently being harvested and industrially processed to an oil product, Calanus^®^ Oil (Pedersen et al., 2014b). *Calanus finmarchicus* is an important prey item for many ecologically and economically important fish species such as herring and mackerel (Prokopchuk and Sentyabov, 2006), and harvesting lower down the food web can have serious impacts on the recruitment and survival of these planktivorous fish species if not done with care. Also, the by-catch of eggs, larvae, and fry during direct catching of *Cf* may have potential negative effects further up in the food chain (Eysteinsson et al., 2018). The Norwegian Directorate of Fisheries has proposed a total catch quota for direct catching of *Cf* of 254 000 tonnes a year (Norwegian Directorate of Fisheries, 2016a). This quota is based on an estimated standing stock of 33 million tonnes in the Norwegian Sea and following similar regulations as for krill (*Euphausia superba*) fishing in the Antarctic (Norwegian Directorate of Fisheries, 2016b). It is argued that the standing stock of *Cf* is so high that no effect of the proposed quota will be seen on the population size. In addition, although the proposed total quota is 254 000 tonnes a year, the actual harvest in 2016 was 660 tonnes (Thorvik, 2017). But nonetheless, the size of the total quota will be re-assessed at a later stage, when increased biological knowledge and more experience from the harvesting activities and catch processing on board is available (Norwegian Directorate of Fisheries, 2016a).

*C. finmarchicus* has a lifespan of only one year (Falk-Petersen et al., 2009) resulting in very low levels of persistent organic pollutants in the lipid fraction (AMAP, 2009; Mizukawa et al., 2009), and refinement of the oil is therefore not necessary. The lipid class composition of the oil from *Cf* is, however, different from other marine oils. In traditional whole-body fish oil and cod liver oil, EPA and DHA are generally bound to a glycerol backbone forming triacylglycerol (TAG). Second-generation n-3 LC-PUFA fish oil supplements have concentrated contents of EPA and DHA, either in the form of ethyl esters or re-esterified TAG. Krill oil is also often included in this group, although it has a high content of phospholipids in addition to TAG (reviewed by Xie et al., 2019). The oil from *Cf* has a unique chemistry, where most of the fatty acids esterify with long-chain fatty alcohols, forming the lipid class known as wax esters (Lee et al., 2006) (**Figure 1**). For this reason, Calanus^®^ Oil may be regarded as a third generation of n-3 products. This new marine oil is, however, a niche product compared to bulk oils, like fish oils. The total fish production globally in 2016 was around 171 million tonnes, of this approximately 20 million tonnes were used for non-food purposes, mostly for the production of fish meal and fish oil (FAO, 2018). This results in almost 1 million tonnes of fish oil (Tocher, 2015). In contrast, the harvest of *Cf* is below 1000 tonnes a year, and with a lipid content of approximately 8% (Falk-Petersen et al., 1987; Scott et al., 2000; Lee et al., 2006), this results in less than 100 tonnes of oil,

The aim of this review is to discuss the possible role of the novel marine oil from *Cf* as a metabolic therapy to prevent obesity-induced low-grade inflammation and the associated metabolic disturbances. However, a challenge with writing this review is the lack of clinical studies with calanus oil, while its potential health benefits are based primarily on animal studies. The unique chemistry of this oil argues, however, for the view that this oil is not just another EPA- and DHA-containing oil. Therefore, we have chosen to discuss the impact of the major components of calanus oil on metabolic health in the light of available information in the literature.

## Biosynthesis of Long-Chain Omega-3 Fatty Acids

**Figure 2:**
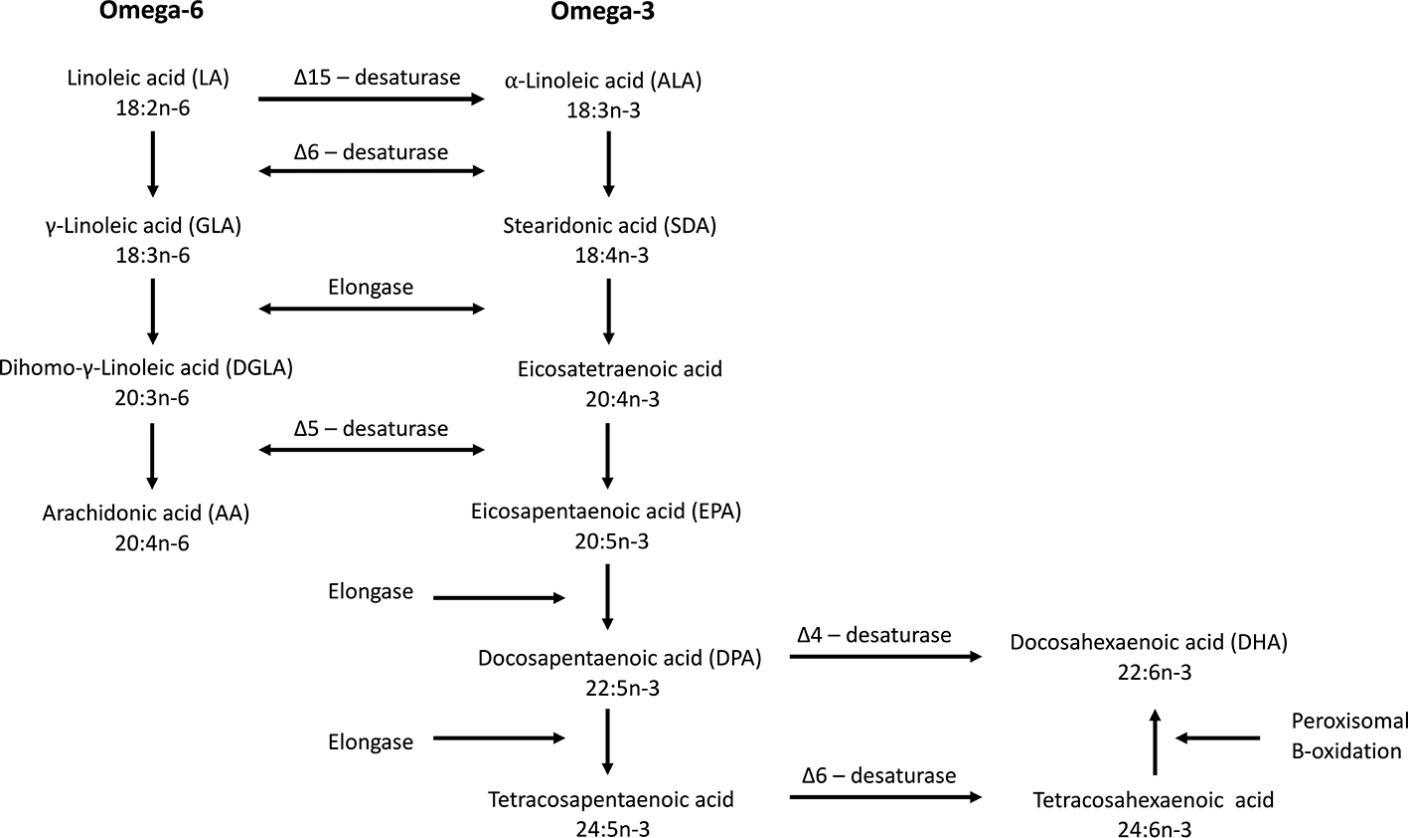
Biosynthesis of omega-6 and omega-3 fatty acids. Δ-15 desaturase is only found in plants. Δ-4 desaturase is present in some vertebrates but not in humans. The Δ-6 desaturase catalyze is the rate limiting step in the synthesis of EPA and DHA (modified from Calder, 2015).

Humans are not able to synthesize n-3 fatty acids *de novo* and, therefore, depend on the diet to obtain them directly, or synthesize them from dietary essential fatty acids such as α-linolenic acid (ALA). *De novo* synthesis of omega-6 and n-3 fatty acids from oleic acid is only possible in plants, including microalgae, because they possess the Δ-12 and Δ-15 desaturases. Delta-12 desaturase produces linoleic acid that can be converted further into ALA by Δ-15 desaturase. Humans can convert linoleic acid into arachidonic acid (AA) and ALA into SDA and EPA due to the enzymatic activity of Δ-6 and Δ-5 desaturases and elongase (**Figure 2**). The conversion of EPA to DHA is possible *via* two different pathways. After conversion of EPA into docosapentaenoic acid (DPA, 22:5n-3), further conversion into DHA can be done by the so-called Sprecher pathway. In this pathway DPA is first elongated (forming tetracosapentaenoic acid; 24:5n-3) followed by a second Δ-6 desaturation (forming tetracosahexaenoic acid; 24:6n-3) and finally chain shortening *via* peroxisomal β-oxidation to DHA (Sprecher et al., 1995). Lower eukaryotes and some vertebrates, even including some mammals, but not humans, can convert DPA directly into DHA by Δ-4 desaturase (**Figure 2**). The Δ-6 desaturase activity is rate-limiting (Bernert and Sprecher, 1975) making the conversion of ALA to SDA inefficient and the conversion further to DHA very limited. More EPA is therefore formed from SDA than from ALA, but it is only slightly further converted to DHA due to the second Δ-6 desaturase step (Leonard et al., 2004; Lee et al., 2016). The endogenous conversion of ALA to EPA and DHA has been reported to be 21% and 9%, respectively, in young women (Burdge and Wootton, 2002). In men, the conversion from ALA to EPA is only between 0.3% and 8% while the conversion from ALA to DHA is below 4% and often undetectable (Emken et al., 1994; Burdge et al., 2002; Burdge et al., 2003; Hussein et al., 2005). It is therefore important to consume EPA and DHA *via* the diet in order to benefit from the health effects provided by these fatty acids.

## Oil Extracted From *Calanus finmarchicus*

The composition of the different lipid classes in the oil in *Cf* changes depending on the copepod life-cycle stage at the time of harvest, season, and location of sampling (Falk-Petersen et al., 1987; Fraser et al., 1989) (see also **Tables 1** and **2**). The industrially produced oil is obtained in the summer when *C. finmarchicus* is in surface waters and has the highest lipid content due to feeding on the blooming phytoplankton (Pedersen et al., 2014b).Throughout its 1-year life cycle, the lipid content and fatty acid composition of *C. finmarchicus* changes, depending on the life stage/season (Kattner and Krause, 1987). In this Calanus^®^ Oil, more than 85% of the lipids consist of wax esters (Pedersen et al., 2014a) (see also **Table 2**). About 11% of the fatty acids (all lipid classes combined) are monounsaturated fatty acids (MUFA), of which cetoleic acid (22:1n-11) and gondoic acid (20:1n-9) are the most abundant with about 4% and 2.5%, respectively, followed by 1.5% oleic acid (18:1n-9). Approximately 19% of the fatty acids are PUFA of which 18% are omega-3 fatty acids. This novel marine oil is relatively low in EPA and DHA compared to other marine oils (6% and 4% respectively), but is relatively rich in SDA (7%), see **Table 2** (Pedersen et al., 2014a; Cook et al., 2016b). The main fatty alcohols present are the equivalents of the dominant LC-MUFA in the oil, namely the monounsaturated long-chain fatty alcohols eicosenol (20:1n-9) and docosenol (22:1n-11) (Pedersen et al., 2014a). In **Table 2**, it can be seen that in oil in *C. finmarchicus* the abundance of EPA and DHA is highest in the phospholipids. The actual amount, however, is low since the proportion of phospholipids is low compared to the neutral lipid classes (**Table 1**). It has been published that the bioavailability of EPA and DHA is higher from the phospholipids than from TAG (Maki et al., 2009; Schuchardt et al., 2011; Ulven and Christiansen, 2015; Cook et al., 2016a). But this remains, however, controversial (Salem and Kuratko, 2014). Phospholipids are, however, not detected in the commercial Calanus^®^ Oil (Pedersen et al., 2014a) probably due to endogenous enzymatic hydrolysis (Vang et al., 2013). The oil has also been reported to contain about 1500 ppm astaxanthin (Pedersen et al., 2014b). This antioxidant has anti-inflammatory and anti-atherogenic potential, which has been extensively studied in both humans and animals (Jacobsson et al., 1999; Jacobsson et al., 2004; Pashkow et al., 2008; Yang et al., 2011a). However, any possible health effects of astaxanthin will not be discussed here.

**Table 1:** Lipid class composition of *C. finmarchicus* sampled in summer, autumn, winter, and spring in Balsfjord, Norway.

**	% Total Lipid			
*Lipid Class:*	June	October	January	March
*Wax esters*	85.4	88.1	90.0	84.9
*Phospholipids*	4.2	7.3	5.7	10.3
*Triacylglycerols*	8.9	1.3	0.8	nd
*Cholesterol*	1.2	2.6	2.4	3.2
*Free fatty acids*	0.2	nd	1.1	1.7

nd, not detected. Source: Falk-Petersen et al. (1987).

**Table 2:** Fatty acid and fatty alcohol composition (mass %) of the commercial Calanus^®^ Oil and in the different lipid classes in *Calanus finmarchicus*.

**	Lipid class				
*Fatty acid*	Calanus^^®^^ Oil^a^	WE^b^	WE^c^	TAG^b^	PL^b^
*14:0 (Myristic)*	6.4	26.3	18.0	12	3.3
*16:0 (Palmitic)*	4.5	9.8	9.3	30.4	25.8
*18:0 (Stearic)*	0.2	0.9	nd	6.1	3.6
*16:1n-7 (Palmitoleic)*	1.7	6.7	6.5	3.6	1.1
*18:1n-9 (Oleic)*	1.6	5.3	5.3	10.4	2.5
*20:1n-9 (Gondoic)*	2.4	7.8	9.6	nd	0.2
*22:1n-9 (Erucic)*	0.3	0.2	nd	nd	nd
*22:1n-11 (Cetoleic)*	4.3	7.0	12.0	2.2	0.2
*18:2n-6 (LA)*	0.7	1.2	nd	2.7	1.5
*18:3n-3 (ALA)*	1.4	1.5	nd	2.3	0.6
*18:4n-3 (SDA)*	7.0	13.7	9.3	5.9	2.5
*20:5n-3 (EPA)*	5.5	11.4	9.8	8.7	19.2
*22:5n-3 (DPA)*	0.3	nd	nd	1.2	0.2
*22:6n-3 (DHA)*	3.9	2.2	7.7	5.8	37.4
***Fatty alcohol***					
*14:0*	0.4	3.9	1.1		
*16:1n-7*	0.5	3.4	1.8		
*18:1n-9*	1.0	nd	nd		
*20:1n-9 (Eicosenol)*	12.9	39.3	41.0		
*22:1n-9*	1.0	nd	nd		
*22:1n-11 (Docosenol)*	18.8	38.8	45.2		

Source: ^a^ Pedersen et al. (2014a), ^b^ Albers et al. (1996) ^c^ Kattner et al. (1989).

The fatty acid values per lipid class presented here are from C. finmarchicus females^b^ and copepod stage V^c^ harvested during the summer in the Fram Strait. Modified from Pedersen et al. (2014b) and Falk-Petersen et al. (2009).

nd, not detected; SFA, saturated fatty acid; MUFA, monounsaturated fatty acids; PUFA, polyunsaturated fatty acids; WE, wax ester; TAG, triacylglycerol; PL, phospholipid.

A complete description of the digestion of wax esters and absorption of fatty acids and fatty alcohols are beyond the scope of this paper. But minor amounts of waxes are present in a variety of food items (Hargrove et al., 2004). The consumption of large portions of wax ester rich fish has been reported to cause outbreaks of keriorrhea “oily diarrhea,” and associated stomach cramps, nausea, and vomiting, in several countries. This has led to the suggestion that wax esters are indigestible (Ho Ling et al., 2009). However, other publications have demonstrated that mammals can digest wax esters, at least when consumed in moderate amounts (Hansen and Mead, 1965; Yaron et al., 1982; Gorreta et al., 2002) As reviewed by Hargrove et al. (2004), humans are able to hydrolyze the waxes found in a variety of food items and absorb the liberated fatty acids and alcohols. The safety of the oil from *Cf* for human consumption has been clinically evaluated by Tande et al. (2016), and there are no safety concerns regarding this novel marine oil when consumed in recommended amounts of 2g of calanus oil. Parallel to this safety trial ran a study on the bioavailability of EPA and DHA in oil from *Cf* for human consumption (Cook et al., 2016b). The volunteers in the study by Cook et al. (2016b) consumed 4g calanus oil without any ill effects.

Feeding experiments in mice have shown that the fatty alcohols present in the oil are detected in the feces of mice, indicating that the wax esters are indeed hydrolyzed. The same study also detected increased incorporation of different n-3 LC-PUFA in liver and white adipose tissue, indicating absorption of wax ester-derived fatty acids (Pedersen et al., 2014a).

Calanus oil is a novel marine oil, and it has only since recently been harvested for production of a nutraceutical. It has earlier been used in feed for farmed Atlantic salmon (*Salmo salar*) (Bogevik et al., 2009) and Atlantic halibut (*Hippoglossus hippoglossus*) (Colombo-Hixson et al., 2013). However, oils from zooplankton are considerably more expensive than fish oils and are, therefore, currently not included in general aquaculture feed. Clinical studies are currently being conducted to examine the ability of oil from *Cf* to combat obesity and insulin resistance, but no results have been released to date. Feeding experiments on rodents, however, have shown that dietary supplementation with only 1% to 2% calanus oil improved metabolic and inflammatory parameters in high-fat diet-induced obese mice (Höper et al., 2013; Höper et al., 2014). The oil has also been reported to attenuate atherosclerotic lesion formation (Eilertsen et al., 2012), reduce hypertension (Salma et al., 2016), and protect the heart from ischemic stress (Jansen et al., 2019). Results from Höper et al. (2014) indicated that supplementation of the diet with purified wax ester has stronger anti-inflammatory and anti-obesogenic effects in diet-induced obese mice, compared to ethyl esters of EPA and DHA. This suggests that not only EPA and DHA, but also other components from the hydrolyzed wax esters in the oil, or the wax ester itself, might have beneficial effects on health. In particular, the observation that even very low dosages of the oil can counteract obesity-induced metabolic dysfunction holds promise that calanus oil could be promising nutraceutical in the future

## Obesity Induced Chronic Low-Grade Inflammation

**Figure 3:**
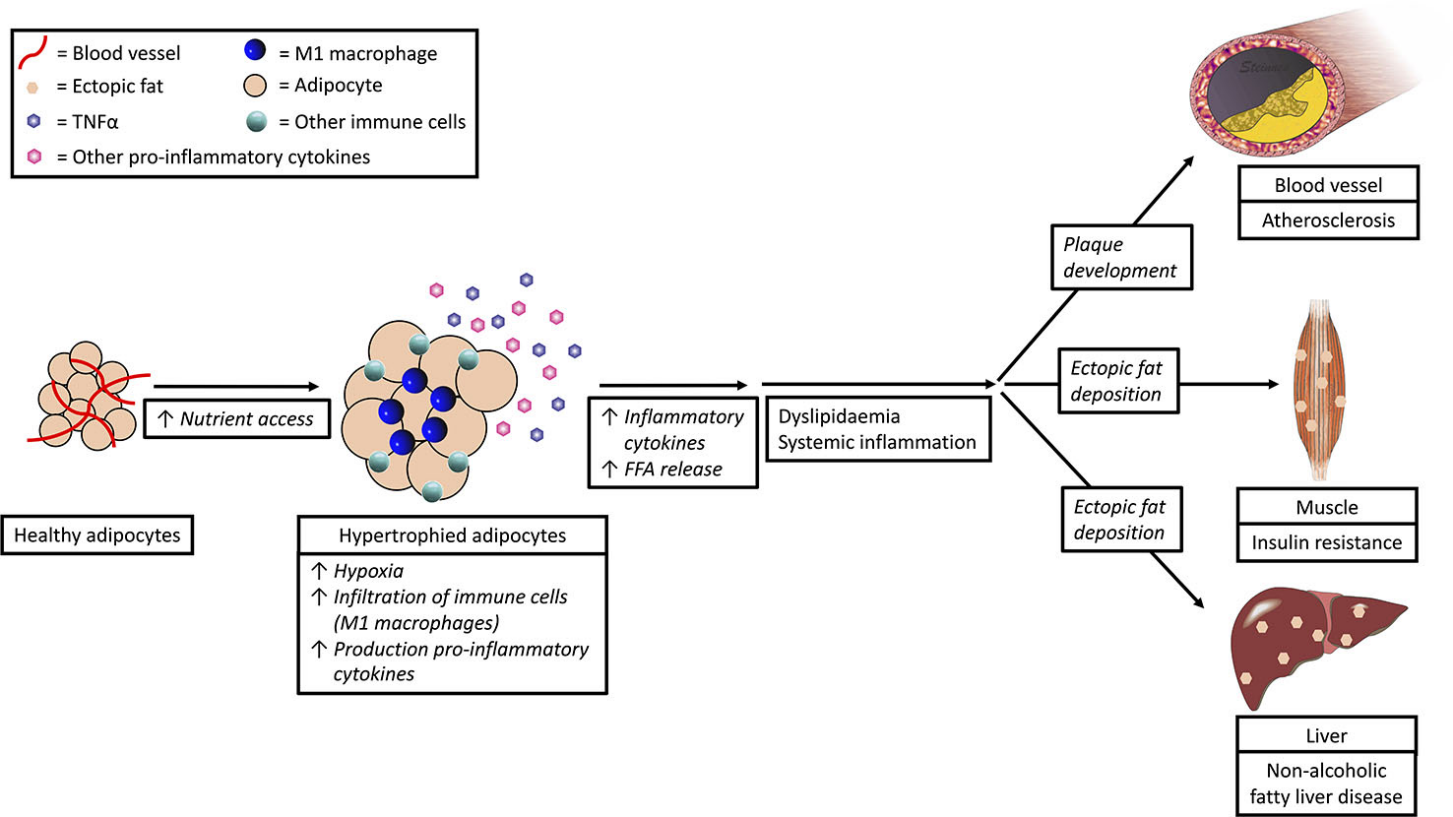
Expansion of adipocytes due to nutrient access leads to dyslipidemia and inflammation and subsequent cardiovascular disease, insulin resistance, and non-alcoholic fatty liver disease.

Obesity, in particular abdominal obesity, is associated with a chronic local low-grade inflammation (Solinas and Karin, 2010; Gregor and Hotamisligil, 2011; Ouchi et al., 2011) with progressive immune cell infiltration in adipose tissue (**Figure 3**). In this process, the immune cells and (to a lesser extend) the enlarged/expanded adipocytes start to secrete pro-inflammatory cytokines (e.g., TNFα, IL-6, and IL-1β) and chemokines, such as monocyte chemoattractant protein-1 (MCP-1) (Fain, 2006).

Numerous studies have shown that hypoxia and nutrient excess are the two main triggering factors for inflammation in adipose tissue (Schenk et al., 2008; Ye, 2009; Gregor and Hotamisligil, 2011). In response to nutrient excess, adipocytes expand and become hypertrophic. At the same time, the distances between the blood vessels increase and oxygen diffusion becomes insufficient (Torres Filho et al., 1994), leading to local hypoxia, which in turn triggers the secretion of cytokines *via* activation of Hypoxia-Inducible Factor (HIF)-1 Alpha (Sun et al., 2013).

Infiltration of pro-inflammatory cells in inflamed adipose tissue is characterized by infiltration of M1 macrophages in replacement of M2 macrophages. M2 macrophages produce anti-inflammatory cytokines such as IL-10 and IL-23 and recruit regulatory T cells. M1 macrophages, however produce pro-inflammatory cytokines such as TNFα and IL-6, and attract Th1 cells (Fujisaka et al., 2009; as reviewed in Mills, 2012). Adipose tissue is the key site of interaction between adipocytes and immune cells due to the architectural organization and proximity of these cell types. Access to blood vessels allows for soluble mediators to communicate with other organs. In this way, the inflammatory status of adipose tissue becomes a risk factor for disease development, including metabolic syndrome, insulin resistance, diabetes mellitus, and cardiovascular disease (Mokdad et al., 2003; Reaven, 2005; Hotamisligil, 2006; Mathew et al., 2008; Van de Voorde et al., 2013) (**Figure 3**).

## The Main Components of the Oil from *Cf* and Their Effect on Inflammation Control

### EPA and DHA

Fish oils have long been considered to promote positive health effects through the n-3 LC-PUFA EPA and DHA (Simopoulos, 1991). Treatment of severely obese non-diabetic patients with EPA and DHA has shown to reduce adipose tissue mass and systemic inflammation (Itariu et al., 2012). An updated meta-analysis of 13 randomized controlled trials, which included over 120 000 participants confirmed that marine n-3 LC-PUFA supplementation reduces the risk for coronary heart disease (CHD) and cardiovascular disease (CVD), myocardial infarction, and death due to CHD and CVD (Rimm et al., 2018; Hu et al., 2019). The American Heart Association concluded, based on new scientific data, that the prescription of n-3 LC-PUFA at a dose of 4 g/day can be used as monotherapy or in combination with other lipid-lowering agents to reduce hypertriglyceridemia (Skulas-Ray et al., 2019). Clinical studies and a recent meta-analysis, including 20 clinical trials, have confirmed therapeutic effects of n-3 LC-PUFA supplementation in rheumatoid arthritis patients (Gioxari et al., 2018; Woodman et al., 2019). Moreover, a systematic review and meta-analysis by Natto et al. (2019) concluded that these omega-3 fatty acids may be associated with lower plasma levels of inflammatory biomarkers in diabetic patients. However, results regarding their effect on glucose metabolism, insulin resistance, and type 2 diabetes are less clear (Stella et al., 2018). Also regarding other chronic diseases, such as non-alcoholic fatty liver disease and chronic kidney disease, the effects of n-3 LC-PUFA are inconclusive (Jump et al., 2018; Saglimbene et al., 2019). Factors that may account for the inconsistent findings regarding the use of n-3 LC-PUFA supplements are the doses used, the choice of placebo, and the duration and type of intervention (El-Bayoumy and Manni, 2020).

**Figure 4:**
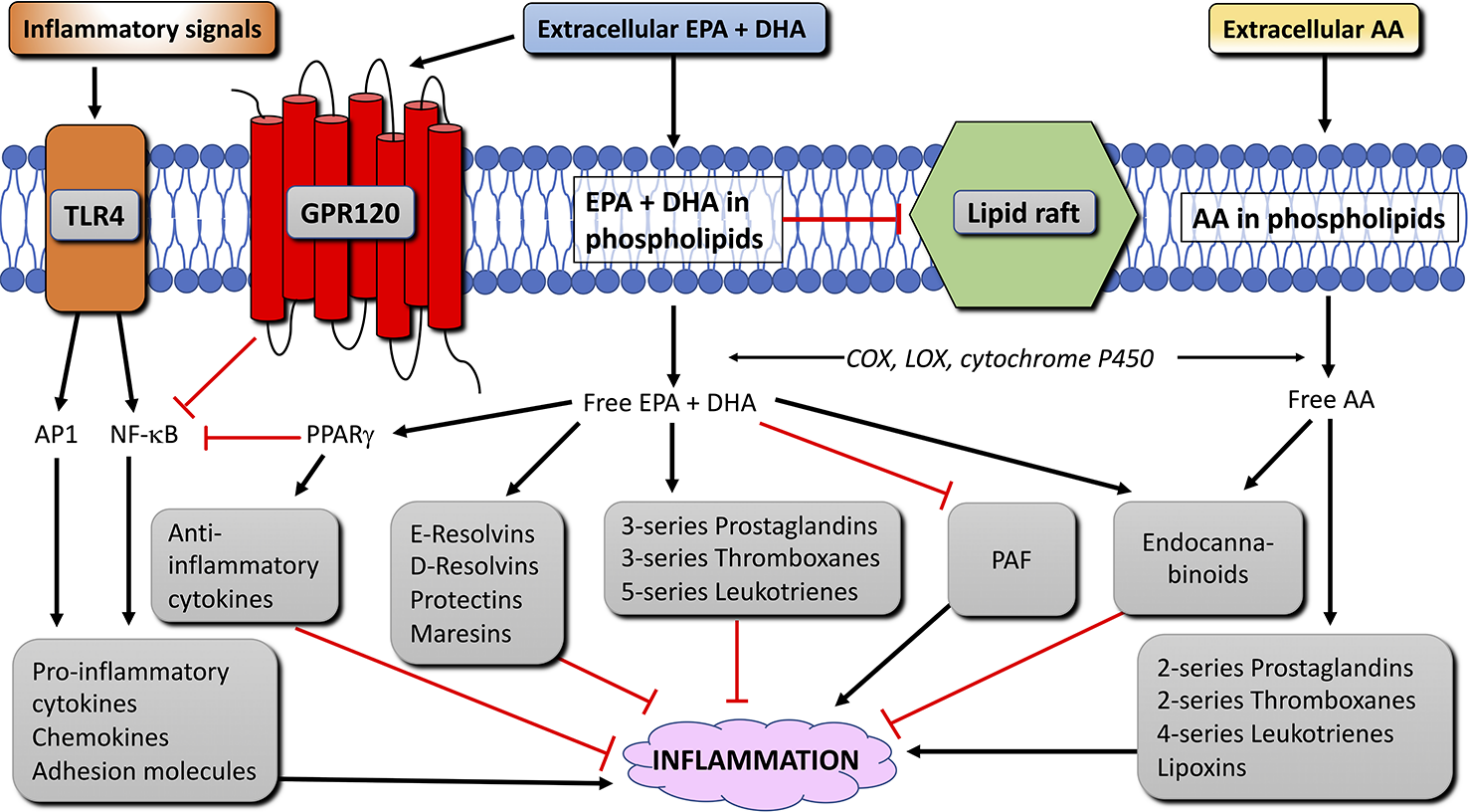
Anti-inflammatory mechanisms and pathways of EPA and DHA in immune cells. Black arrow lines indicate activation, red blunted lines indicate inhibition. AA, arachidonic acid; AP1, activation protein 1; COX, cyclooxygenase; DHA, docosahexaenoic acid; EPA, eicosapentaenoic acid; GPR120, G-protein receptor 120; LOX, lipoxygenase; NFκB, nuclear factor κ B; PAF, platelet-activating factor; PPAR, peroxisome proliferator activated receptor; TLR4, toll-like receptor 4 (modified from Calder, 2015).

Although the benefits of consuming n-3 LC-PUFA may remain controversial for some diseases and conditions, it is well accepted that n-3 LC-PUFA have anti-inflammatory effects. These anti-inflammatory effects and their possible mechanisms have been extensively reviewed by Calder (2015) and Rogero and Calder (2018). The two main mechanisms are changes in the phospholipid composition of the cell membrane and changes in the activation of pro- and anti-inflammatory transcription factors (**Figure 4**) and their target genes.

#### Alterations of the Membrane Phospholipid Composition of Immune Cells

Intake of n-3 LC-PUFA alters the fatty acid composition of the membrane phospholipids of immune cells, affecting the production of AA- and EPA-derived eicosanoids from the phospholipids. AA is a major substrate for the enzymes cyclooxygenase (COX), lipoxygenase (LOX) and cytochrome P450. Enzymes that catalyze the metabolisms of AA to 2-series prostaglandins and thromboxanes and 4-series leukotrienes and lipoxins that mainly serve as pro-inflammatory lipid mediators (reviewed in Calder, 2009). EPA is a substrate for the same enzymes, and competes with AA as a substrate for COX and LOX. EPA-derived eicosanoids form 3-series prostaglandins and thromboxanes and 5-series leukotrienes. These EPA-derived eicosanoids have been regarded less potent than those produced from AA, and thereby stimulate inflammation to a lesser extent (Goldman et al., 1983; Lee et al., 1984; Bagga et al., 2003; Wada et al., 2007; Tull et al., 2009). Calder (2009), pointed out, however, that not all AA-derived eicosanoids (e.g. prostaglandin E2) are pro-inflammatory.

#### Production of Lipid Mediators That Resolve Inflammation

The incorporation of n-3 LC-PUFA in the cell membrane increases the production of lipid mediators that resolve inflammation, namely, the EPA-derived E-series resolvins and the DHA-derived D-series resolvins, protectins, and maresins (**Figure 4**). These lipid mediators were shown to have anti-inflammatory and protective properties both in cell culture and animal models of inflammatory diseases, and aid in the resolution of the inflammation (2008a; Serhan et al., 2008b; Bannenberg and Serhan, 2010; Serhan and Chiang, 2013; Balas et al., 2014). In addition to eicosanoids and pro-resolving lipid mediators, phospholipids containing n-3 (and n-6) LC-PUFA can also become metabolized to produce endocannabinoids. The endocannabinoids can bind to cannabinoid receptor types 1 and 2, which have anti-inflammatory properties (Artmann et al., 2008; Batetta et al., 2009; Wood et al., 2010). Other studies have shown that through incorporation in the cell membrane, n-3 LC-PUFA reduce the production of platelet-activating factor in certain immune cells (Croft et al., 1986; Pickett et al., 1986; Sperling et al., 1987; Shikano et al., 1993; Martin–Chouly et al., 2000; Watanabe et al., 2001). Finally, incorporation of EPA and DHA disrupts the formation of lipid rafts in the membrane, which can lead to changes in cell signal transduction during inflammation (Reviewed by Pike, 2003).

#### Long-Chain Omega-3 Fatty Acid-Induced Activation of Transcription Factors

Nuclear factor kappa B (NF-κB) is a transcription factor that up-regulates genes encoding pro-inflammatory cytokines, adhesion molecules, chemo-attractants, and enzymes needed to produce eicosanoids. Toll-like receptor 4 (TLR4) is a membrane protein that, upon activation, initiates a signaling pathway that activates NF-κB and the transcription factor activator protein 1 (AP1), leading to increased inflammation (**Figure 4**). In adipose tissue of obese people, TLR4 signaling triggers chronic low-intensity inflammation (reviewed in Rogero and Calder, 2018). Long-chain omega-3 fatty acids, however, limit the activation of TLR4 by inhibiting the translocation of TLR4 to lipid rafts, due to the disrupting effect these fatty acids have on raft formation (Rogero and Calder, 2018). Long-chain omega-3 fatty acids further reduce the inflammatory effect of NF-κB and AP1 by binding to G-protein coupled receptor 120 (GPR120) (Oh et al., 2010; Oh and Olefsky, 2012). GPR120 is highly expressed in adipocytes, the distal part of the intestine, and in macrophages (Gotoh et al., 2007; Oh et al., 2010). GPR120 activation in macrophages inhibits activation of NF-κB and thereby reduces inflammation (Oh et al., 2010; Li et al., 2013; Yan et al., 2013; Williams-Bey et al., 2014) (**Figure 4**). Peroxisome proliferator-activated receptor (PPAR)-γ is a transcription factor expressed in immune cells and adipocytes. PPAR*γ* can be activated by omega-3 fatty acids and physically interfere with NF-κB and thereby decreasing inflammation (**Figure 4**). Furthermore, PPAR*γ* can form a heterodimer with retinoid-X-receptor (RXR). Both PPAR-*γ*:RXR as well as PPAR*γ* and RXR alone are transcription factors for anti-inflammatory mediators and can be activated by PUFA and lipid mediators produced from AA, EPA, and DHA (Forman et al., 1997; Kliewer et al., 1997; de Urquiza et al., 2000; Desreumaux et al., 2001; Vanden Berghe et al., 2003; Szanto and Nagy, 2008; Zapata-Gonzalez et al., 2008). Finally, due to the disruption of membrane rafts and its associated intracellular signaling by EPA and DHA, n-3 LC-PUFA also inhibit T cell responses (Stulnig and Zeyda, 2004; Yaqoob, 2009; Kim et al., 2010). Thus, EPA and DHA have anti-inflammatory effects *via* several mechanisms. The interaction between these fatty acids and other cellular components in immune cells is illustrated in **Figure 4**.

To sum up, it appears that n-3 LC-PUFA can attenuate diet-induced obesity and inflammation *via* several mechanisms. In light of the fact that calanus oil contains relatively small amounts of these particular fatty acids, it is likely that it is not only EPA and DHA which are responsible for the anti-obesogenic and anti-inflammatory effects of the oil, but other components in addition. Of note, these effects were obtained with a low supply (1.5%) of calanus oil (Höper et al., 2013; Höper et al., 2014), whereas Mori et al. (2007) reported reduced adiposity in mice fed a high-fat diet supplemented with fish oil (containing 44.8% DHA and 5.9% EPA) only when the content of fish oil reached 8%.

### Stearidonic Acid

SDA is the Δ-6 desaturation product of ALA (**Figure 2**). Oil from *Cf* contains about 7% SDA (Pedersen et al., 2014a; Cook et al., 2016b), which is high compared to fish oils (McGill and Moffat, 1992). SDA is naturally present at about 12% in echium oil and at 20% in *Buglossoides arvensis* oil, also called Corn gromwell (Bimbo et al., 2013). In addition, it has now been developed gene-modified soybeans in which SDA is enriched. The SDA content in such soybean oil is about 20% (Wilkes, 2008). The main effect of SDA on inflammation control appears to be dependent on its conversion to EPA, while little is known about any direct effects. Supplementation with SDA-containing oil to the diet increases the abundance of EPA in blood lipids, peripheral blood mononuclear cell, other immune cells, red blood cells, and in heart tissue in both humans and animals (James et al., 2003; Miles et al., 2004a; Miles et al., 2004b; Harris et al., 2007).

Although the EPA concentration is often increased upon SDA consumption, any subsequent health effects are not clear (Banz et al., 2012; Deckelbaum et al., 2012; Whelan et al., 2012; Walker et al., 2013). SDA supplementation trials with overweight or slightly obese human volunteers have shown inconclusive results regarding the effect on the omega-3 index, which may be an important measure for the risk of developing CVD and insulin resistance (Burrows et al., 2011). Pieters and Mensink (2015) did not find any effect of SDA on the omega-3 index despite an increase in EPA in the red blood cell membranes, whereas other studies did confirm an increase in the omega-3 index (Harris et al., 2008; Lemke et al., 2010). Supplementation of SDA-rich oil does not appear to have an effect on TAG, total cholesterol (TC), low-density lipoprotein cholesterol (LDL-C), and high-density lipoprotein cholesterol (HDL-C) concentrations in plasma of healthy, overweight or slightly obese humans (Harris et al., 2008; Whelan, 2009; Lemke et al., 2010; Krul et al., 2012; Pieters and Mensink, 2015). However, the lipid profile was improved in lean and obese Zucker rats fed SDA-enriched soybean oil (Casey et al., 2013).

Also, regarding inflammation, the health benefits of SDA supplementation remain unclear. There was no effect on the production of TNFα and IL-1β in LPS-stimulated whole blood from healthy volunteers (James et al., 2003). Miles et al. (2004b) observed that SDA can increase the EPA status in immune cells, but did not observe an effect on human immune function. While Hsueh et al. (2011) found a reduced IL-6 secretion in LPS-stimulated adipose stem cells from *ob/ob* mice due to a suppressed TLR2 expression and a decreased activity of NF-κB. SDA also downregulated the levels of inducible nitric oxide synthesis (iNOS) protein, the translocation of NF-κB and the phosphorylation of mitogen-activated protein kinases (MAPK) in LPS-induced (M1) macrophages (Sung et al., 2017).

SDA does not increase the abundance of DHA in humans, due to the second Δ-6 desaturation step (**Figure 2**). This might be one of the reasons why SDA does not appear to have a clear effect on human health. Other reasons can be the low doses used or the duration of the experiment. Also, as with EPA and DHA, the chemical structure (TAG, ethyl ester or wax ester) might play a role.

Although the effect of SDA on immune function and lipid profile remains inconclusive, SDA is one of the most potent fatty acids for activating GPR120 (Kotarsky et al., 2003; Christiansen et al., 2015). GPR120 is highly expressed in macrophages and adipocytes. The two cell types that play a crucial role in obesity and the development of the underlying chronic inflammation and metabolic syndrome. As previously stated, GPR120 activation in macrophages has anti-inflammatory effects. GPR120 activation in adipocytes stimulates adipocyte differentiation (Gotoh et al., 2007; Miyauchi et al., 2009) and improves insulin sensitivity and enhances glucose uptake due to an increased translocation of glucose transporter 4 (GLUT4) from the cytosol to the cell membrane (Talukdar et al., 2011). In murine models, the expression of GPR120 in adipose tissue is induced by thermogenic activation, promoting browning of white adipose tissue and brown fat activation. Browning of white adipose tissue is an important component of energy expenditure and can lead to weight loss (Quesada-López et al., 2016; Sharma et al., 2019). GPR120 activation is reported to improve glucose tolerance, insulin resistance, and chronic inflammation in obese mice and is, therefore, a target for the treatment of obesity and type 2 diabetes (Oh et al., 2010; Ichimura et al., 2012; Oh et al., 2014; Yore et al., 2014).

The diverse tissue distribution of the GPR120 may indicate several functions related to systemic metabolism and inflammation. Recently, more attention has been given to the anti-inflammatory role of this receptor in intestinal cells (Anbazhagan et al., 2016). It has been shown that the expression of GPR120 and other free fatty acid receptors (GPR40 and GPR119) are more abundant in the lower intestine, especially in the colon (Hirasawa et al., 2005; Miyauchi et al., 2009; van der Wielen et al., 2014). However, dietary lipids, such as TAG and phospholipids are quickly digested and absorbed in the upper parts of the gastrointestinal system and will normally not reach the lower intestine (Carey et al., 1983). In contrast, wax esters in calanus oil are more hydrophobic than dietary TAG and, therefore, more difficult to emulsify. In addition, other enzymes than those hydrolyzing TAG and phospholipids are probably involved. As a result, wax esters may exhibit a longer retention time to facilitate hydrolysis and absorption (Cowey and Sargent, 1977; Verschuren and Nugteren, 1989). The wax esters may therefore act as a natural delayed release of potent stimulators of GPR120, such as SDA, EPA, and DHA in this part of the intestinal system. Of interest, ongoing studies aim at developing systems of nutrient delivery to pass beyond the proximal small intestine by using a form of enteric coating to obtain a delayed release of the potent GPR120 agonists (Sørensen, 2018).

The health benefit of SDA is not clear, but the mechanism may partly depend on its conversion to EPA, so that it acts indirectly *via* the mechanisms described in section *EPA and DHA*. In addition, the ability of SDA to activate GPR120 receptors on macrophages, adipocytes, and intestinal cells could be another mechanism by which SDA could alleviate obesity-induced inflammation. Both mechanisms seem plausible in light of the high content of SDA in calanus oil and should be followed up by new mechanistic studies.

### Monounsaturated Fatty Acids

About 10% of the fatty acids in calanus oil are monounsaturated fatty acids, of which cetoleic acid (22:1 n-11) and gondoic acid (20:1 n-9) are the most abundant, with approximately 4% and 2.5%, respectively (Pedersen et al., 2014a; Cook et al., 2016b). The interest for monounsaturated fatty acids in a health perspective is mainly based on the observation that the incidence of chronic diseases is relatively low among the adult population in certain regions bordering the Mediterranean Sea. Olive oil, which is a major component of the Mediterranean diet, is rich in oleic acid (18:1n-9), and the health-promoting effect of the diet has to some extent been ascribed to this MUFA (Delgado-Lista et al., 2016). Dietary MUFA have been associated with cardio protection (Pérez-Jiménez et al., 2002) and reduction of risk factors for development of metabolic syndrome (reviewed in by Gillingham et al., 2011). The replacement of saturated fatty acid (SFA) with MUFA (18:1n-9) in the diet may improve the blood lipid profile by lowering TAG, TC, and (V)LDL-C, while preserving HDL-C. In addition, replacement of SFA with MUFA improves body composition and insulin sensitivity while reducing hyperglycemia and hypertension in individuals predisposed to metabolic syndrome (reviewed in Gillingham et al., 2011).

More recently, the role of long-chain mono-unsaturated fatty acids (LC-MUFA), having aliphatic chains of more than 18 C atoms, has been studied (reviewed by Yang et al., 2016b). These fatty acids are found in high amounts in many fish oils (McGill and Moffat, 1992). Oil from *Cf* is rich in the LC-MUFA, cetoleic acid (22:1n-11), and a recent report indicated that this particular fatty acid may improve the efficiency of the conversion of ALA to EPA and DHA (Østbye et al., 2019).

Dietary intake of different marine oils rich in LC-MUFA (in addition to n-3 LC-PUFA) reduces the risk factors of metabolic syndrome in animal models by improving plasma lipid levels and insulin sensitivity (Østerud et al., 1995; Lindqvist et al., 2009; Yang et al., 2011b; Yang et al., 2011a; Yang et al., 2015). Saury and herring oil have been reported to decrease adipocyte size and cause an increase in n-3 LC-PUFA levels and a concomitant decrease in n-6/n-3 PUFA ratio in different tissues (Lindqvist et al., 2009; Yang et al., 2011b; Yang et al., 2015). Herring oil, and seal oil combined with olive oil, have been found to reduce atherosclerotic lesions in the aorta (Eilertsen et al., 2011; Gabrielsson et al., 2011). Saury oil is also reported to reduce hepatic TC (Yang et al., 2011c) and TAG content (Yang et al., 2011c; Yang et al., 2015). Furthermore, saury and pollock oil increased plasma adiponectin levels, and decreased plasma levels of resistin, leptin, (Yang et al., 2011c; Yang et al., 2011b) and TNFα (Yang et al., 2011b) in animal models.

**Figure 5:**
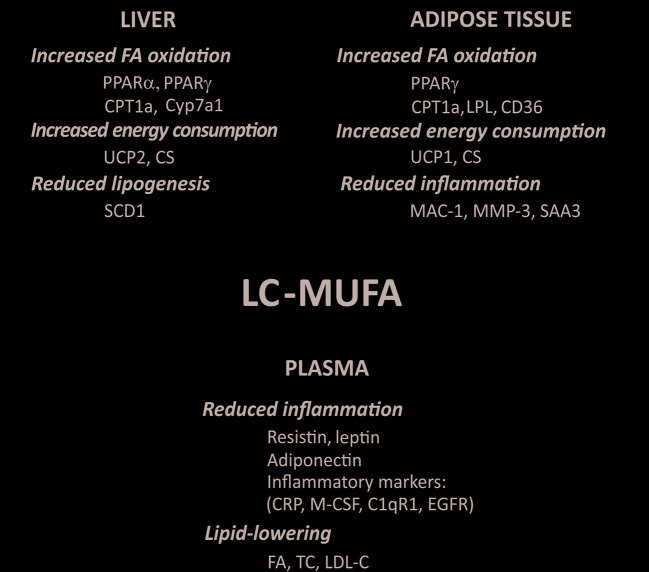
Reported effects of concentrated LC-MUFA on gene expression related to fatty acid oxidation, energy consumption, lipogenesis, and inflammation in the liver and adipose tissue, as well as plasma levels of various compounds. C1qR1, complement component 1q receptor; CD36, fatty acid translocase; CPT1a, carnitine palmitoyltransferase 1a; CRP, C-reactive protein; CS, citrate synthase; CyP7a1, cholesterol 7 alpha-hydroxylase (cytochrome P450 7A1); EFGR, epidermal growth factor receptor; FA, free fatty acids; LDL-C, low-density lipoprotein cholesterol; LPL, lipoprotein lipase; MAC-1, macrophage-1 antigen; M-CSF, macrophage colony-stimulating factor; MMP-3, matrix metalloproteinase-3; PPAR, peroxisome proliferator-activated receptor; SAA3, serum amyloid A3; SCD-1, stearoyl-CoA desaturase-1; TC, total cholesterol; UCP, uncoupling protein (summarized from Yang et al., 2011d; Yang et al., 2013; Yang et al., 2015; Yang et al., 2016a; Yang et al., 2017).

The marine oils used in the studies mentioned above are all rich in LC-MUFA. However, they also contain n-3 LC-PUFA, and it is therefore not possible to attribute the observed biological effects solely to the LC-MUFA component in the various oil preparations. A few studies have, however, used diets supplemented with concentrated LC-MUFA to investigate the effects attributed specifically to this class of fatty acids. This type of experimental design has investigated the health effects of LC-MUFA on atherogenesis, obesity-induced inflammation, glucose and lipid metabolism, and the expression of associated genes in different animal models (Halvorsen et al., 2001; Yang et al., 2011d; Yang et al., 2013; Yang et al., 2015; Yang et al., 2016a; Yang et al., 2017). From these studies, it can be concluded that LC-MUFA decrease atherosclerotic lesion formation, reduce cholesterol efflux and alter gene expression related to inflammation, lipid metabolism and energy expenditure in different tissues (**Figure 5**). It appears that the carbon chain length of the dietary MUFA can be an important factor that determines its metabolic effects. For instance, LDL receptor knock out (LDLR-KO) mice fed a Western diet enriched with 2% LC-MUFA concentrate displayed suppressed levels of aorta atherosclerotic lesions and plasma inflammatory markers such as C-reactive protein (CRP), macrophage-colony stimulating factor (MCSF) and complement component 1q, receptor 1(C1qR1). These effects were not observed when the mice were fed the same diet enriched with 2% oleic acid-rich olive oil when compared to control (Yang et al., 2016a).

Although chain length might be an important factor, the exact mechanisms behind the health promoting and cardioprotective effects of LC-MUFA are not fully understood. However, LC-MUFA are considered ligands of PPARs (Grygiel-Górniak, 2014). Thus, LC-MUFA concentrate supplementation has been shown to increase the expression of *pparγ* and its target genes, and decreased inflammatory marker expression in white adipose tissue (Yang et al., 2013). This was associated with reduced adipocyte size. LC-MUFA have also been reported to decrease atherosclerosis *via* PPAR signaling (Yang et al., 2016a; Yang et al., 2017). *Pparα,* *pparγ,* and their target genes *Cyp7a1* (encoding cholesterol 7α-hydroxylase or bile acid synthase) and *Adipor2* (gene for adiponectin 2 receptor) were reported to be upregulated in the liver of the LC-MUFA fed mice (Yang et al., 2016a). Over-expression of the CYP7A1 enzyme protects against atherosclerosis (Miyake et al., 2002) and adiponectin improves metabolic syndrome and atherosclerosis (Hui et al., 2012).

As mentioned, other studies using concentrated LC-MUFA (Yang et al., 2011d) or LC-MUFA rich fish oil (Yang et al., 2011b; Yang et al., 2011c; Yang et al., 2015) have reported that these fatty acids stimulate expression of other genes involved in inflammation, lipid metabolism and insulin signaling (see also **Figure 5**). However, the direct involvement of the PPAR signaling pathway in the expression of each of these genes is not fully described. Further studies are needed to elucidate if LC-MUFA exert beneficial health effects *via* mechanisms other than the proposed PPAR signaling pathways, and if some of these direct mechanisms distinguish the effects of LC-MUFA from those of LC n-3 PUFA.

### Fatty Alcohols

The most abundant fatty alcohols in calanus oil are the monounsaturated fatty alcohols docosenol (22:1 n-11) and eicosenol (20:1 n-9) (**Table 2**). The health promoting properties of fatty alcohols became an area of interest when researchers in Cuba (Mas et al., 1999; Castaño et al., 2001; Arruzazabala et al., 2002) reported beneficial effects of policosanol from sugarcane wax on the plasma lipoprotein profile (increased HDL-C and reduced TC and LDL-C). Policosanol is a mixture of essential very-long-chain fatty alcohols with carbon backbones longer than 22 C (Juturu and Gormley, 2013). The effects reported by the Cuban researchers were ascribed to the unique composition of the fatty alcohols from Cuban-derived sugarcane wax. It was suggested that the cholesterol lowering effect of policosanol was due to inhibition of HMG-CoA reductase synthesis following hepatic conversion of fatty alcohols to their corresponding fatty acids (Menéndez et al., 2001).

Research groups outside Cuba have long failed to reproduce and validate the efficiency of policosanol in improving the lipoprotein profile (as reviewed by Marinangeli et al., 2010). However, a recent meta-analysis including 13 Cuban and 9 non-Cuban studies confirmed the efficacy and safety of sugarcane policosanol on dyslipidemia (Gong et al., 2018). In addition, experimental studies with Cuban policosanol in rats (Cho et al., 2018b), as well as clinical studies in healthy Korean subjects (Kim et al., 2017; Cho et al., 2018a; Kim et al., 2018) showed reduced body fat (Kim et al., 2017; Cho et al., 2018a) and improved blood lipid profile (Kim et al., 2017; Cho et al., 2018a; Cho et al., 2018b; Kim et al., 2018). It has been reported that this was due to inhibition of cholesteryl ester transfer protein (Kim et al., 2017).

Sugarcane wax is not the only source of (very) long-chain fatty alcohols that have been tested on human health. Montserrat-de la Paz et al. (2014) and Fernández-Arche et al. (2009) studied the anti-inflammatory effects of long-chain fatty alcohols from evening primrose oil and pomace olive oil, respectively. They showed that long-chain alcohols from both pomace olive oil and primrose oil inhibited TNFα and nitric oxide production in LPS-stimulated murine (M1) macrophages in a dose-dependent manner through inhibition of inducible nitric oxide synthase (iNOS). Pomace olive oil also decreased the production of the pro-inflammatory mediators prostaglandin E2 (PGE_2_), in murine macrophages, and thromboxane B2 (TXB_2_) in rat peritoneal neutrophils. Reduced release of these eicosanoids was due to inhibition of secretory phospholipase A2 (sPLA_2_) (Fernández-Arche et al., 2009). The long-chain fatty alcohols from evening primrose oil had no effect on PGE_2_ formation, but did cause a dose-dependent inhibition of the secretion of sPLA_2,_ TXB_2_, and IL-1β in LPS-stimulated (M1) macrophages (Montserrat-de la Paz et al., 2014). The fatty alcohols from evening primrose oil also reduced the gene expression of cyclooxygenase-2, the enzyme needed for the production of eicosanoids, in a dose dependent manner (Montserrat-de la Paz et al., 2014).

Tetracosanol from sugarcane wax was shown to improve glycemic control *via* activation of insulin receptor kinase and translocation of GLUT 4 from the cytosol to the plasma membrane (Hsu et al., 2015). Recently, the health-promoting effects of policosanol and octacosanol have received new interest. Guo et al. (2017) showed that octacosanol improves the health status in a mouse model of colitis by reducing pathological damage in colonic tissue and inhibiting the gene and protein expression levels of TNFα, IL-1β, IL-6, and iNOS in the colon. Octacosanol also reduced the gene and protein expression of these pro-inflammatory cytokines in LPS-stimulated (M1) macrophages. Sharma et al. (2019) reported that policosanol and octacosanol supplementation reduced body fat gain, decreased insulin resistance, and reduced hepatic lipid content in high-fat diet-induced obese mice. This was associated with increased thermogenesis in brown adipose tissue due to GPR120 activation, as well as decreased expression of genes involved in lipogenesis and cholesterol uptake in the liver and reduced inflammation in white adipose tissue. Classical studies in rats have shown that fatty alcohols may be oxidized to their corresponding fatty acids (Stetten and Schoenheimer, 1940; Blomstrand and Rumpf, 1954) in the endoplasmic reticulum during hepatic metabolism and subsequent chain shortening in the peroxisomes (Hargrove et al., 2004). The effects of policosanol and octacosanol supplementation found by Sharma et al. (2019) were suggested to be due to the conversion of these fatty alcohols to their corresponding fatty acids.

Policosanols occur in different natural products (Shen et al., 2019; Weerawatanakorn et al., 2019). The fatty alcohols eicosenol (20:1n-9) and docosenol (22:1n-11) found in the wax esters in calanus oil are shorter in chain-length, compared to the different fatty alcohols in policosanols. It is therefore difficult to extrapolate the health effects of the policosanols mentioned above to those in oil from *Cf*. However, Hsu et al. (2015) found that chain length of the policosanols did not affect their impact on glycemic control. Thus, it might be that the fatty alcohols found in calanus oil have similar effects as the policosanols.

Previous studies in our lab with calanus oil have demonstrated incorporation of the mono-unsaturated fatty acids, 20:1n-9 (in white adipose tissue) and 22:1n-11 (in liver) of mice fed a high-fat diet supplemented with the oil (Pedersen et al., 2014a). This could reflect the content of LC-MUFA in the oil, but also the *in vivo* oxidation of the corresponding fatty alcohols. This leads to the suggestion that the calanus oil-induced health effects may not be entirely due to the fatty acids, but indirectly also to the fatty alcohols in the oil. Calanus oil differs from other marine oils in terms of its content of fatty alcohols, and conversion of these alcohols to their corresponding monounsaturated fatty acids could boost the uptake of these specific fatty acids. The quantitative importance of this intriguing mechanism, as well as its metabolic implications, needs to be determined in new studies.

## Conclusion

The oil from *Calanus finmarchicus* is a marine oil with a unique chemistry. Although relatively low in EPA and DHA, it contains high amounts of SDA and a number of monounsaturated fatty acids. The fatty acids are bound to long-chain fatty alcohols forming wax esters that constitute approximately 85% (w/w) of the oil. The various classes of fatty acids, as well as the fatty alcohols may have potential health benefits, since it is likely that fatty alcohols are oxidized to the corresponding fatty acids after absorption. This review has focused on the effect of the various components of calanus oil in relation to prevention of chronic low-grade inflammation, but more research is needed to determine the efficacy of the various components in this respect, or whether an anti-inflammatory effect of the oil is a result of the combined action of several components.

## Author Contributions

PS drafted the manuscript. PS, AP, K-EE, RO, and TL critically revised and edited the manuscript for clarity and content.

## Funding

This work was supported by UiT The Arctic University of Norway (The MarVal Project). The publication charges for this article have been funded by a grant from the publication fund of UiT The Arctic University of Norway.

## Conflict of Interest

TL has a small position as scientific advisor in Calanus AS. AP is employed as product manager by Calanus AS.

The remaining authors declare that the research was conducted in the absence of any commercial or financial relationships that could be construed as a potential conflict of interest.

## Abbreviations

AA, arachidonic acid; ALA, α-linolenic acid; *Cf, Calanus finmarchicus;* DHA, docosahexaenoic acid; DPA, docosapentaenoic acid; EPA, Eicosapentaenoic acid; GPR120, G-protein coupled receptor 120; HDL-C, high-density lipoprotein cholesterol; LC-MUFA, long-chain monounsaturated fatty acids; n-3 LC-PUFA, Omega-3 long-chain polyunsaturated fatty acids; LDL-C, low-density lipoprotein cholesterol; NF-κB, nuclear factor kappa B; PPAR, peroxisome proliferator-activated receptor; SDA, stearidonic acid; SFA: saturated fatty acid; TAG, triacylglycerol; TC, total cholesterol; TLR4, Toll-like receptor 4.
